# *cmv1*-Mediated Resistance to CMV in Melon Can Be Overcome by Mixed Infections with Potyviruses

**DOI:** 10.3390/v15091792

**Published:** 2023-08-23

**Authors:** Andrea Giordano, Inmaculada Ferriol, Juan José López-Moya, Ana Montserrat Martín-Hernández

**Affiliations:** 1 Centre for Research in Agricultural Genomics (CRAG) CSIC-IRTA-UAB-UB, Edifici CRAG, Campus UAB, Bellaterra, 08193 Barcelona, Spain; andrea.giordano@cragenomica.es (A.G.); iferriol@ica.csic.es (I.F.); juanjose.lopez@cragenomica.es (J.J.L.-M.); 2 Institut de Recerca i Tecnologia Agroalimentàries (IRTA), Edifici CRAG, Campus UAB, Bellaterra, 08193 Barcelona, Spain

**Keywords:** cucumovirus, potyvirus, genetic resistance, mixed infections, melon

## Abstract

Resistance to cucumber mosaic virus (CMV) strain LS in melon is controlled by the gene *cmv1*, which restricts phloem entry. In nature, CMV is commonly found in mixed infections, particularly with potyviruses, where a synergistic effect is frequently produced. We have explored the possibility that this synergism could help CMV-LS to overcome *cmv1*-mediated resistance. We demonstrate that during mixed infection with a potyvirus, CMV-LS is able to overcome *cmv1*-controlled resistance and develop a systemic infection and that this ability does not depend on an increased accumulation of CMV-LS in mechanically inoculated cotyledons. Likewise, during a mixed infection initiated by aphids, the natural vector of both cucumoviruses and potyviruses that can very efficiently inoculate plants with a low number of virions, CMV-LS also overcomes *cmv1*-controlled resistance. This indicates that in the presence of a potyvirus, even a very low amount of inoculum, can be sufficient to surpass the resistance and initiate the infection. These results indicate that there is an important risk for this resistance to be broken in nature as a consequence of mixed infections, and therefore, its deployment in elite cultivars would not be enough to ensure a long-lasting resistance.

## 1. Introduction 

Plant viruses are considered responsible for about half of the emerging infectious diseases in plants [1]. In nature, plant viruses are frequently found in mixed infections produced by two or more viruses. However, our perception of this situation is biased because mixed infections are only reported and studied in cases when the combination synergistically exacerbates the symptoms caused by either one of the viruses individually or when other quantifiable effects, such as increases in their titers, are characterized [2]. Considering the virus–virus interactions during mixed infections will help us to understand the evolution and ecological dynamics of plant viruses [3], and taking mixed infections into account could be highly relevant for managing resistance against viral plant pathogens.

*Cucumber mosaic virus* (CMV, genus *Cucumovirus*, family *Bromoviridae*) is able to infect more than 1200 plant species [4]. This ability is provided by its great genetic variability, with a large number of strains that are divided into two subgroups with about 70% nucleotide homology between them: subgroup I (further subdivided into IA and IB) and subgroup II (SGII) [5]. Its genome is composed of three positive single-stranded RNAs (RNA1, RNA2 and RNA3). RNA1 and RNA 2 encode proteins 1a and 2a, which are involved in replication, and 2b, which is involved in RNA silencing suppression. RNA3 encodes 3a, the movement protein (MP), and 3b, the coat protein (CP). In nature, CMV is transmitted by aphids in a non-persistent manner, with the viral CP being the major determinant of the process [6]. In melon, resistance to CMV in the Korean accession PI 161375, which is the cultivar Songwhan Charmi (SC), is oligogenic and recessive [7] and composed of one gene, *cmv1*, which provides full resistance to SGII CMV strains, such as CMV-LS, and at least two QTLs, which together with *cmv1* provide resistance to SGI strains such as CMV-M6 and CMV-FNY [8,9]. Experiments using a near isogenic line (NIL) that carried only one introgression from SC including *cmv1* in the background of the susceptible melon cultivar Piel de Sapo (PS), demonstrated that CMV-LS can replicate and move cell-to-cell but remains restricted to the bundle sheath (BS) cells, providing resistance to phloem entry and impeding the systemic infection [10]. This restriction is dependent on the viral movement protein (MP), since both CMV-FNY and a mutant CMV-LS carrying FNY MP can overcome this resistance [11]. *Cmv1* acts as a general gatekeeper for phloem entry, not only in the accession SC but in all other melon resistant varieties previously tested [12]. This gene encodes vacuolar protein sorting 41 (VPS41) [13], a protein involved in the intracellular transport of cargo proteins from the late Golgi to the vacuole, as part of the homotypic fusion and vacuole protein sorting (HOPS) complex [14]. VPS41 supports CMV infection in the susceptible melon PS cultivar by organizing transvacuolar strands that could be used by the virus for its movement towards the plasmodesmata (PD). These strands are nearly absent in the resistant SC accession [15]. In plants, *Arabidopsis thaliana* VPS41 is involved in pollen–stigma interaction and mutants in this protein are frequently sterile [16]; however, is also involved in vegetative growth [17].

Resistance to CMV-FNY phloem entry in SC is occasionally overcome so that the virus can produce a mild infection in one or two leaves, depending on the environmental conditions, although it will never produce a systemic infection. Indeed, during a preliminary coinfection of CMV-FNY and the potyvirus zucchini yellow mosaic virus strain AG II (ZYMV-AG II), we observed that the former was able to systemically infect SC, whereas CMV-FNY alone was unable to do so (not shown). The known synergistic effect between cucumoviruses and potyviruses [18,19,20,21] and the available information concerning the two viruses suggests that their co-infections can affect resistances and even result in a modification of the viral distribution within plant tissues [22,23]. Thus, it was reasonable to think that CMV could be accumulating more in the coinfected plants than in the single-infected ones, leading to the hypothesis that during co-infections CMV could more efficiently use the few transvacuolar strands present in the BS cells of the resistant accession SC to reach the phloem and develop a systemic infection. This hypothetical scenario would imply a risk for resistance management, in which the deployment of *cmv1* into elite cultivars would not avoid CMV damage during mixed infections. In this study, we have explored this hypothesis using a simpler system, the NIL SC12-1-99, which only carries the gene *cmv1*, instead of using SC, which carries more than three resistance QTLs [9]. We have also compared CMV mechanical inoculations, which deliver a larger input of virus into wounds, with insect-mediated inoculations, which are more similar to infections in nature and have the potential to inoculate fewer virions but do so directly into different plant tissues.

## 2. Materials and Methods

### 2.1. Plant, Insects and Virus Materials

The melon (*Cucumis melo*) genotypes used in this study were the Korean accession PI 161375, cultivar Songwhan Charmi, the Spanish type Piel de Sapo, and the NIL SC12-1-99, which carries an introgression of SC on the linkage group XII that contains the *cmv1* gene [8]. Seeds were pre-germinated and grown as described by Guiu-Aragonés et al. [11].

A clonal population of the aphid *Myzus persicae*, designated MP89 and originally from the ICA-CSIC (Madrid, Spain), was maintained on tobacco plants (*Nicotiana tabacum* ‘Xanthi’).

The cucumovirus strain CMV-LS was provided by Professor P. Palukaitis from Seoul’s Women’s University (Korea) as infectious clones of the three genomic RNAs [24]. The potyviruses ZYMV-AGII (attenuated strain) and watermelon mosaic virus LL2B3 (WMV-LL2B3) infectious clones were provided by Prof. A. Gal-On from Volcani Center (Israel) [25] and Dr. C. Desbiez from INRAE (France) [26], respectively.

### 2.2. Viral Inoculations

For CMV-LS inoculations, cotyledons of 7 to 10-day-old melon plants were inoculated either mechanically or using aphids as vectors. For rub inoculation, sap freshly obtained from infected zucchini squash Chapin F1 (Semillas Fitó S.A., Barcelona, Spain) was used as described in [11]. For aphid transmission, experiments were performed essentially as described in [27]. Apterous adult individuals were collected, starved for 2 h in glass vials and allowed to acquire the virus on a symptomatic infected leaf for 10 min, before being transferred manually to the receptor plants with a fine paintbrush until the desired number of 10 viruliferous aphids were placed on each individual test plant for the inoculation period. Vector aphids were killed by spraying the plants with Confidor insecticide (imidacloprid 0.08%, Bayer, Leverkusen, Germany). In mixed infections, either cotyledons or the first leaf were inoculated with CMV-LS and WMV-LL2B3 or CMV-LS and ZYMV-AGII. Individual inoculations with CMV-LS, ZYMV-AGII or WMV LL2B3 were used as controls. For the mixed infection with CMV-LS and ZYMV-AG II, cotyledons of 7 to 10-day-old melon plants were agroinfiltrated with *Agrobacterium* C58C1 harboring ZYMV-AGII and rub-inoculated with the fresh sap of CMV-LS after two days post-infiltration (dpi). A visual phenotyping was performed at 15 and 30 dpi using a 6 degrees scale varying from 0 to 5 according to the severity of symptoms as previously reported [9]. For the mixed infection with CMV-LS and WMV-LL2B3, vector-mediated inoculations were performed with viruliferous aphids for each one of the individual viruses.

### 2.3. Sampling and RNA Extraction

Samples from inoculated cotyledons were collected after five dpi and leaf discs from the youngest fully expanded leaf was collected at 15 or 30 dpi. Samples were immediately frozen in liquid nitrogen and ground for RNA extraction using TRIzol^TM^ reagent (Invitrogen, Carlsbad, CA, USA) according to the manufacturer’s instructions, including an additional ethanol precipitation to improve purity. RNA quality was assessed through gel electrophoresis and quantification was assessed using NanoDrop ND-8000 spectrophotometer. Samples were treated with TURBO-DNAse I (Applied Biosystem, Ambion, CA, USA) following manufacturer’s instructions and 1 μg of RNA was used for cDNA synthesis using the SuperScript III Reverse Transcriptase Kit (Invitrogen, Carlsbad, CA, USA) and random primers (Invitrogen, Carlsbad, CA, USA) following the manufacturer’s instructions.

### 2.4. Quantification of CMV Viral Load by RT-qPCR

For CMV quantification, absolute RT-qPCR with the standard curve method was used. To generate the standard RNA curve, a fragment of 1.5 kb located near the 5′ ends of the viral genomic RNA3 was cloned on pGEMT-Easy (Promega, Madison, WI, USA) under the control of the T7 promoter. Transcripts were obtained with linearized plasmids as templates using the MEGAscript T7 kit (Ambion, Austin, TX, USA) and a five-point standard curve from 10-fold dilutions was built to use as standard.

RT-qPCR was performed on a LightCycler 480 Real-Time PCR System (Roche Applied Science, Indianapolis, IN, USA) using SYBR Green I Mix (Roche Applied Science, Indianapolis, IN, USA). Cycling conditions were as follows: 10 min at 95 °C, 40 cycles of 95 °C for 15 s, 20 s at 60–65 °C and 72 °C for 20 s, followed by a melting curve cycle from 65 to 95 °C. CMV primers (F: CCGGTGAATTGCGCTCTAAA, R: CAAAGAACCCTCAGCATCCG) were designed using Primer BLAST. The presence of secondary structure was checked with Oligo Calculator version 3.27 (http://biotools.nubic.northwestern.edu/OligoCalc.html, accessed on 4 June 2020). The primer specificity was tested by PCR amplification and agarose gel electrophoresis. For each experiment, three or four biological replicates and two technical replicates were used.

### 2.5. Virus Detection by Reverse Transcriptase-PCR

RT-PCR was performed using Superscript III RT (Invitrogen Life Technologies, Carlsbad, CA, USA) and Taq polymerase (Promega Corporation, Madison, WI, USA) according to manufacturer’s instructions. The primers used for detection of CMV were CMV_F: GTTTTATTTACAAGAGCGTACG and CMV_R: GAAGCATTCCACATATCGTAC, which amplify a 1400 nt fragment, and for detection of WMV were WMV_F: AGCAAAGGATCTTTTGGCTATG and WMV_R: CACTCACAAAGTTTCTTGAATATG, which amplify a 269 nt fragment.

## 3. Results

### 3.1. Mixed Infections Allow CMV-LS to Overcome cmv1-Controlled Resistance without Increasing Its Accumulation in the Inoculated Cotyledons

The mixed infections by CMV-LS and a potyvirus were studied in melon cultivar Piel de Sapo (PS), which is susceptible to CMV; the Korean accession PI 161375, cultivar Songwhan Charmi (SC), which is resistant to CMV; and the NIL SC12-1-99, which carries an introgression of SC containing the *cmv1* resistance gene [8].

**Figure 1:**
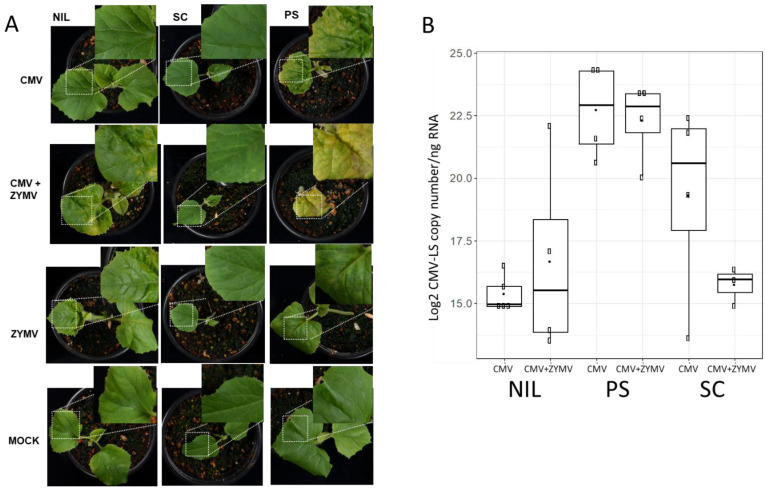
Assessment of viral infection in mechanically inoculated single and mixed infections. (**A**) Plants of genotypes PS, SC and NIL SC12-1-99 (NIL) at 15 dpi in either single (CMV-LS or ZYMV-AGII) or mixed (CMV-LS + ZYMV-AGII) infections. All plants are at the same developmental stage after germination. Characteristic symptoms of the plants are amplified for one representative individual plant per each group. (**B**) Boxplot depicting the accumulation of CMV-LS viral RNA (Log2 CMV-LS copy number/ng of total RNA) in inoculated cotyledons at 5 dpi. Viral RNA was detected by RT-qPCR. The data are the average of three biological replicates. Boxplot represents the median and interquartile range (IQR).

Groups of 10 plantlets corresponding to the different plant materials were used. In a first experiment, symptoms resembling those caused by CMV infections, such as leaf curling, yellowing and mosaic in the leaves, were observed at 15 dpi in two out of seven surviving NIL SC12-1-99 plants after double-inoculation, whereas as expected, no symptoms were shown in those inoculated with only CMV-LS (nine plants). Meanwhile, the PS controls were fully infected both in single and mixed infections (6 and 10 plants, respectively). The mixed infected PS controls showed stronger symptoms, whereas all SC plants showed normal growth and development on both single and mixed infections (10 plants in each case). Single ZYMV-inoculated PS and NIL 12-1-99 plants showed mild mosaic corresponding to ZYMV symptoms, whereas SC showed just a mild vein clearing. Pictures of the symptoms observed in representative plants of the different categories are shown in Figure 1A. A replica of the experiment was performed, this time reaching 7 out of 10 NIL SC12-1-99 plants with symptoms after double-inoculation, and at the same time, a higher mortality for PS-infected plants was observed. Thus, CMV-LS was able to overcome *cmv1*-mediated resistance in at least some of the plants when infecting in the presence of the potyvirus ZYMV. To test if an increase in the CMV-LS amount in the inoculated cotyledons could be accountable for the breakage of resistance, the levels of CMV-LS were quantified in samples taken from cotyledons after 5 dpi (Figure 1B) and also in newly developed leaves at 15 dpi for selected plants (Supplementary Table S1), both in single and mixed infections, in order to monitor the infection during plant growth. No increase in CMV-LS accumulation was detected in the inoculated cotyledons in the mixed infections compared with the single CMV-LS-infected ones (Figure 1B). Thus, although the presence of ZYMV did not lead to an increase in CMV-LS accumulation in the inoculated cotyledon, *cmv1*-mediated resistance was overcome. Therefore, this indicates that there is no threshold of CMV-LS accumulation above which the virus can overcome *cmv1* resistance in the NIL SC12-1-99. The synergistic interaction of both viruses was associated with an increase in CMV-LS accumulation in systemically infected leaves of PS. However, in the case of the mixed infection in the NILSC12-1-99 after 15 dpi, despite suffering an evident visual systemic infection, no correlation was observed between viral load and the presence of symptoms, whereas in the resistant accession SC, CMV-LS was under the limit of detection in the equivalent samples (Supplementary Table S1).

### 3.2. cmv1 Resistance Is Compromised in Mixed Infections Initiated by Aphid Inoculation

Mechanical inoculation is considered to require a viral input higher than the amount transmitted by aphids, which are the usual transmission vectors of CMV in nature. To know if the aphid-transmitted CMV-LS would be able to overcome *cmv1*-mediated resistance after mixed infection with a potyvirus, we also performed an approach that simulates the infection in nature by aphids. As the clone ZYMV-AGII has a disabled HC-Pro gene [28,29], it could not be used for this experiment. Thus, WMV strain LL2B3 was used as a co-infecting potyvirus instead. For this approach, 10 plants from NIL SC12-1-99, PS and SC were inoculated either with CMV-LS alone or in combination with WMV using 10 viruliferous aphids per plant and per virus (therefore, 10 + 10 = 20 aphids for the co-inoculations of CMV + WMV).

**Figure 2:**
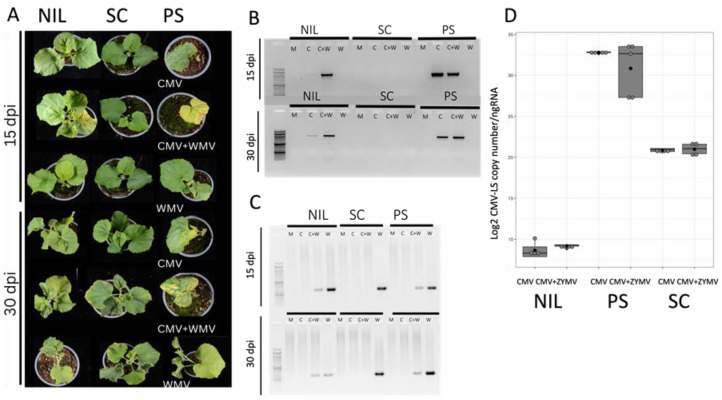
Assessment of viral infection in single and mixed infections inoculated by aphids. (**A**) Plants of genotypes PS, SC and NIL SC12-1-99 at 15 and 30 dpi of either single (CMV-LS or WMV-LL2B3) or mixed (CMV-LS + WMV-LL2B3) infections, as indicated. (**B**) CMV detection by RT-PCR in newly developed leaves at 15 and 30 dpi for mock (M) single (C or W) and mixed infections (C + W). (**C**) WMV detection by RT-PCR in newly developed leaves at 15 and 30 dpi for mock (M), single (C or W) and mixed infections (C + W). (**D**) Boxplots representing the accumulation of CMV-LS RNA particles (log2 CMV-LS copy number/ng of total RNA) of CMV-LS in the inoculated cotyledons at 5 dpi. The data are the average of three biological replicates. Boxplots represent the median and interquartile range (IQR).

Mild to moderate mosaic symptoms were observed in the NIL SC12-1-99 at 15 dpi in the coinfected plants that were not present in the single CMV-LS- or WMV-inoculated plants, indicating that using aphid inoculation the potyvirus allowed CMV-LS to overcome *cmv1*-mediated resistance. This was confirmed by RT-PCR detection, showing a CMV-LS specific band in the plants with mixed infection (Figure 2A,B). At 30 dpi, 80% of the plants presented moderate symptoms in the mixed infection and again, RT-PCR demonstrated that CMV-LS was present in these systemically infected leaves. In this case, the CMV-inoculated plant shown in Figure 2B shows a faint band corresponding to CMV, suggesting that some CMV-LS had managed to invade the phloem, which is a situation that occurs very scarcely. Furthermore, the presence of detectable CMV did not correlate with symptoms of viral infection. At this stage, single WMV-infected NIL SC12-1-99 and PS plants showed mild curly leaves and mosaic. Neither CMV symptoms nor RT-PCR bands corresponding to CMV were detected in SC plants, either in single or mixed infection, whereas those plants produced a systemic WMV infection (Figure 2A,C). Interestingly, the quantification of CMV-LS in the aphid-inoculated leaves of the NIL SC12-1-99 showed, again, that equivalent amounts of virus were present in the single and mixed inoculations and that it was barely detectable in both cases (Figure 2D), again suggesting that a higher accumulation of CMV-LS was not a requirement to overcome *cmv1*-mediated resistance. Thus, a CMV-LS input from the inoculating aphids (likely a very small amount of infective virus) when inoculating together with a potyvirus would be enough to establish an infection in natural conditions, overcoming *cmv1*-mediated resistance. However, even higher viral accumulation in the inoculated leaf of SC did not produce a systemic infection in this accession, suggesting that the co-infection with a potyvirus does not allow CMV to overcome the resistance mediated by all QTLs present in SC genome. PS plants showed symptoms of CMV at 15 dpi both in a single infection and when combined with WMV. However, in the mixed infection, more severe symptoms were detected and only half of the plants had survived at 30 dpi. Correspondingly, both CMV and WMV were detected by RT-PCR (Figure 2A,B). SC did not show CMV symptoms, but WMV-related symptoms were evident, such as curly leaves at 30 dpi in the single infection. More interestingly, WMV accumulated at lower levels in the coinfected NIL SC12-1-99 and in PS and was absent from the SC co-infected plants, which suggests a negative interaction for WMV in the presence of CMV in all cases that is stronger in SC (Figure 2C).

Altogether, these results demonstrate that CMV-LS inoculated by aphids in a mixed infection with a potyvirus can establish a systemic infection in the *cmv1*-carrying plants, and this result does not correlate with a higher accumulation of CMV in the mixed infection plants.

## 4. Discussion

Our results indicate that CMV-LS can infect resistant melon plants carrying the gene *cmv1* when co-infecting with a potyvirus, whereas it is unable to overcome *cmv1*-controlled resistance during a single infection. The preliminary results suggested that this could be due to the synergistic effect already reported between CMV and potyviruses, where CMV accumulation could be promoted by the potyvirus [18,30]. However, quantification of CMV-LS present in the inoculated cotyledons after 5 days post-inoculation showed a similar amount of CMV-LS in both the single- and co-infected NIL plants. Thus, an increased virus replication to reach a minimum threshold was not necessary to overcome *cmv1* resistance, suggesting that the synergism between the viruses must be acting at another level independent of CMV replication. In fact, during a single CMV-LS infection, local infection is not restricted either in the NIL or in SC, since in both CMV-LS is able to replicate and move cell-to-cell to reach the veins. However, it is restricted in the BS cells and is unable to move to phloem cells [10]. Thus, the mechanism by which the potyvirus helps CMV-LS to overcome *cmv1*-controlled resistance could be related either to the intracellular movement within the BS cells or to increasing its ability to cross the PD facing the phloem cells. In fact, the interface between the BS and phloem is a boundary for systemic movement in the case of other viruses, such as tobacco mosaic virus in tobacco plants [31], cowpea chlorotic mottle virus infecting soybean plants [32], red clover mosaic virus in *Nicotiana spp*. [33] and for systemic movement of CMV in transgenic tobacco plants expressing the CMV replicase gene [34]. Likewise, during a normal infection in melon PS, a low amount of CMV-LS invading the phloem cells was observed compared with the virus present in BS cells, although it was sufficient to establish a systemic infection [10].

Aphids transmit viruses by inoculating on any part of the leaf where they are probing, including the blade and sometimes the phloem itself and, with potyviruses, they can succeed inoculating an amount ranging from 1 to 300 pl of fluid [35,36]. Unfortunately, there are no equivalent measurements for CMV, a tripartite virus, with its genome packaged into three separated particles. In this case, it is likely that a larger volume will be needed to assure delivery of the three components in the same cell, or in functionally complementing nearby cells, to initiate a productive infection focus. It is important to realize that for multipartite viruses, an accurate quantification of viral load should consider the “genome formula”, representing the relative proportion of each individual component [37,38]. Our experiments demonstrated that when the resistant NIL SC12-1-99, carrying *cmv1* gene, was inoculated by viruliferous aphids, the virus transmitted was able to produce a systemic infection only when CMV-LS was co-inoculated with a potyvirus, in this case WMV. Despite the occasional detection of the presence of CMV (faint band in Figure 2B), in our conditions none of the single CMV-LS-inoculated SC12-1-99 plants resulted in systemic infection. Given that *cmv1*-controlled resistance takes place at the level of phloem entry, it could have been overcome by the aphid probing directly into the phloem; this could have happened during the single CMV infection as well as during the mixed infection. However, systemic infection was only produced upon mixed inoculation, suggesting that it was not achieved by the aphid directly reaching the phloem but, again, was related to the presence of the potyvirus and not related to over-replication of CMV after the double inoculation. In fact, both WMV and ZYMV, as well as other viruses including CMV, use many host proteins to complete their life cycle (for a review see [39]). To invade the phloem, CMV needs to use VPS41, a protein involved in the secretory pathway that transports cargo proteins from the trans Golgi network (TGN) to the vacuole. ZYMV and WMV use vacuolar protein sorting 4, an AAA+ ATPase that is part of the endosomal sorting complexes required for transport (ESCRT) complex and involved in endo membrane trafficking during the endosomal and secretory pathways [40,41]. When CMV is unable to use the VPS41 allele present in the NIL SC12-1-99, the presence of the potyvirus might enable CMV to use an alternative protein to be transported to the PD facing the phloem to develop a systemic infection. CMV-FNYD2b mutants, lacking the 2b gene, were unable to invade the phloem in tomato plants, becoming confined to the BS cells; this restriction was removed when co-infecting with the potyvirus PVY [30]. This supports the idea that the potyviruses can complement CMV’s inability to trespass the BS–phloem boundary when CMV is missing the 2b gene or when wild-type CMV is inoculated into a plant missing a host factor essential for this step, such as VPS41 in the melon NIL SC12-1-99. In this last case, the inability of CMV-LS to invade the phloem was mapped to the MP, since a chimeric CMV-LS virus carrying CMV-FNY MP was able to invade the phloem to establish a systemic infection [11]. Thus, more research is needed to know how potyviruses can support phloem invasion by a mutant CMV-FNYD2b virus or in a plant able to restrict this step.

Our experiments showed that accumulation of WMV was reduced or eliminated in both resistant and susceptible melon types during the mixed infection with CMV-LS, suggesting that the interaction between these two viruses mainly resulted in positive synergism for CMV and not for WMV. In partial agreement with this observations, tomato plants co-infected with CMV-FNY and PVY had lower levels of PVY than single PVY infections at 28 dpi, whereas CMV-FNY was enhanced in the co-infected plants. However, at 60 dpi, PVY increased in the presence of CMV-FNY, reflecting a dynamic situation [30]. Likewise, a decrease in the potyvirus was observed in mixed infections of tobacco infected with CMV and PVY [42] and in cucumber infected with ZYMV and CMV [43]. These apparent contradictions might respond to adaptative changes, in which key elements could be changes in the genome formula of the multipartite CMV [44]. Further research would be needed to determine if CMV exhibits such plasticity in different hosts during mixed infections.

Overall and in practical terms, the results obtained using aphid inoculation indicated that the deployment of the resistance given by *cmv1* into elite cultivars would not be enough to ensure a long-lasting resistance and that deployment of the other QTL present in the accession SC should be needed for a more durable strategy of control when other viruses might be present.

## Data Availability

No new data were created.

## Supplementary Materials

The following supporting information can be downloaded at: https://www.mdpi.com/article/10.3390/v15091792/s1, Table S1: CMV viral detection by RT-qPCR and symptoms in non-inoculated, newly developed leaves from inoculated melon plants at 15 dpi from the two replica experiments.
